# Demographic Characteristics Associated With Perceptions of Personal Utility in Genetic and Genomic Testing: A Systematic Review

**DOI:** 10.1001/jamanetworkopen.2023.10367

**Published:** 2023-05-05

**Authors:** Emily G. Miller, Jennifer L. Young, Anoushka Rao, Eliana Ward-Lev, Meghan C. Halley

**Affiliations:** 1 Stanford Center for Biomedical Ethics, Stanford University School of Medicine, Stanford, California; 2 Center for Genetic Medicine, Northwestern University Feinberg School of Medicine, Chicago, Illinois

## Abstract

**Importance:**

The expansion of genetic and genomic testing in health care has led to recognition that these tests provide personal as well as clinical utility to patients and families. However, available systematic reviews on this topic have not reported the demographic backgrounds of participants in studies of personal utility, leaving generalizability unclear.

**Objective:**

To determine the demographic characteristics of participants in studies examining the personal utility of genetic and genomic testing in health care.

**Evidence Review:**

For this systematic review, we utilized and updated the results of a highly cited 2017 systematic review on the personal utility of genetics and genomics, which identified relevant articles published between January 1, 2003, and August 4, 2016. We also used the original methods to update this bibliography with literature published subsequently up to January 1, 2022. Studies were screened for eligibility by 2 independent reviewers. Eligible studies reported empirical data on the perspectives of patients, family members, and/or the general public in the US on the personal utility of any type of health-related genetic or genomic test. We utilized a standardized codebook to extract study and participant characteristics. We summarized demographic characteristics descriptively across all studies and by subgroup based on study and participant characteristics.

**Findings:**

We included 52 studies with 13 251 eligible participants. Sex or gender was the most frequently reported demographic characteristic (48 studies [92.3%]), followed by race and ethnicity (40 studies [76.9%]), education (38 studies [73.1%]), and income (26 studies [50.0%]). Across studies, participants disproportionately were women or female (mean [SD], 70.8% [20.5%]), were White (mean [SD], 76.1% [22.0%]), had a college degree or higher (mean [SD], 64.5% [19.9%]), and reported income above the US median (mean [SD], 67.4% [19.2%]). Examination of subgroups of results by study and participant characteristics evidenced only small shifts in demographic characteristics.

**Conclusions and Relevance:**

This systematic review examined the demographic characteristics of individual participants in studies of the personal utility of health-related genetic and genomic testing in the US. The results suggest that participants in these studies were disproportionately White, college-educated women with above-average income. Understanding the perspectives of more diverse individuals regarding the personal utility of genetic and genomic testing may inform barriers to research recruitment and uptake of clinical testing in currently underrepresented populations.

## Key Points

**Question:**

What are the demographic characteristics of individuals participating in research on the personal utility of genetic and genomic testing?

**Findings:**

In this systematic review of 52 studies with 13 251 eligible participants, sex or gender was the most frequently reported demographic characteristic, followed by race and ethnicity, education, and income. Participants were disproportionately women or female (71%), White (76%), had a college degree or higher education (65%), and reported income above the US median (67%).

**Meaning:**

These findings suggest that the current literature on the personal utility of genetics and genomics underrepresents the perspectives of individuals with diverse demographic backgrounds.

## Teaser

This systematic review examines demographic characteristics of participants in research studies on the personal utility of genetic and genomic testing in health care.

## Introduction

As the clinical use of genetic and genomic testing grows, clinicians and researchers are increasingly recognizing that, in addition to clinical utility, these tests have the potential to provide what is referred to as personal utility.^1^ While assessment of clinical utility is traditionally focused on observable changes in clinical care or health outcomes (eg, medical management, reduction in morbidity or mortality) and is typically assessed by the clinician, the concept of personal utility acknowledges that certain outcomes may have value for the individuals receiving or seeking these tests even if they do not directly impact their medical care.^2^ Although the concept itself is still evolving, recent reviews on the topic suggest that key dimensions of personal utility include psychosocial benefits to both patients and family members, facilitated social support, improved care coordination, and information to inform reproductive and other health care decision-making, among others.^1,3^

As interest in the topic of personal utility has grown, researchers have employed various methods to define and measure this emerging construct in a range of clinical contexts, including cancer,^4^ prenatal testing,^5^ newborn screening,^6^ direct-to-consumer testing,^7^ and rare disease diagnosis,^8^ among others. While the literature on personal utility is rapidly expanding, the extent to which perspectives of individuals from diverse demographic backgrounds are included in defining and measuring this emerging concept remains unclear. The need to diversify genomic data sets is widely recognized,^9^ but the parallel challenge of including diverse participants in studies of the perceived value of genetic and genomic testing remains relatively unexplored. Understanding diverse perspectives regarding the personal utility of genetics and genomics could help improve recruitment of diverse populations for clinical genomics research^10^ and increase uptake of clinical genetic testing among currently underrepresented demographic groups.^11^

Although previous reviews have described the literature on personal utility, these studies have not reported the demographic characteristics of participants included in the reviewed studies.^1,3^ To address this gap, we conducted a systematic review of the peer-reviewed literature focused on defining and measuring the personal utility of genetic and genomic testing to determine the underlying demographic characteristics of study participants.

## Methods

For this systematic review, relevant studies were identified by (1) using the bibliography of a widely cited 2017 systematic review of the literature by Kohler et al^1^ on the personal utility of genetics and genomics and (2) conducting a new literature search using the original methodology of Kohler et al to identify relevant articles published subsequent to their original review. Here, we describe the methodology used to update the original review by Kohler et al as well as methods for data extraction for all articles and analysis of the synthesized data.

Our methods were designed to comply with the Preferred Reporting Items for Systematic Reviews and Meta-Analyses (PRISMA) reporting guideline whenever relevant.^12^ A preregistered protocol was not used, as our methods followed those of a published study.^1^ Because this study was a systematic review and did not involve human participant research, the Stanford University Institutional Review Board deemed it exempt from review, and the need for informed consent was waived.

### Search Strategy

To update the list of relevant studies from Kohler et al,^1^ we first updated their original search strings to account for changing terminology over time (eg, including *perceived* as well as *personal* utility). We then implemented the authors’ original methodology, which included utilization of 2 complementary search strings to query each of the following 4 databases: PubMed, Scopus, Web of Science, and Embase. The first search string was designed to search both titles and abstracts for terms and phrases related to genetic testing combined with those related to personal utility. The second search string utilized population-focused key words (eg, *personal*, *patient*, *individual*) combined with outcomes-focused key words (eg, *utility*, *value*).

This strategy was originally developed and updated with support from a professional resource librarian and was designed to identify relevant articles that did not use the exact phrases for personal utility without adding excessive false positives to the search results. Finally, we manually reviewed the reference lists of all included articles, as well as any relevant reviews identified through our search, for potentially relevant articles (eTable 1 in Supplement 1 provides the full search strings).

### Screening and Eligibility

Search results were imported into Covidence^13^ for screening, which also removed obvious duplicates. Two independent reviewers (E.G.M. and M.C.H.) then screened all articles based on title and abstract, with all discrepancies automatically moved to full-text screening. Then 2 individuals (E.G.M., J.L.Y., A.R., and/or M.C.H.) reviewed the full text of the remaining studies. Any disagreements were discussed by the entire study team with inclusion determined by consensus.

Studies were eligible if they met the following inclusion criteria: (1) reported empirical data on the perspectives of patients, their family members, or members of the general public; (2) focused on any clinical genetic or genomic test, including pharmacogenomic, single gene, prenatal, or exome or genome sequencing, among others; (3) provided empirical data on participants’ perspectives of the utility of genetic or genomic tests; (4) conducted with participants located in the US; (5) published in English in full-text form; and (6) included in the review by Kohler et al^1^ or published between August 5, 2016, and January 1, 2022. The articles in this systematic review therefore included those published from January 1, 2003, to January 1, 2022. For inclusion criterion 1, articles including multiple participant types (eg, health care clinicians and patients) were included, but only demographic data on eligible participants were included in analysis. Studies that did not disaggregate demographics based on participant type were excluded. For criterion 2, studies of genetic testing for nonhealth purposes (eg, ancestry) were excluded. For criterion 3, the concept of utility has multiple disciplinary origins in fields as varied as philosophy, medicine, and health economics.^2^ To be consistent with the original methodology of Kohler et al,^1^ we define *personal utility* broadly as the perceived value of a genetic or genomic test from the perspective of those who have received or might receive genetic or genomic testing. We therefore did not limit (nor did we specifically tailor) our search based on disciplinary orientation, but we did require explicit use of the term *utility* by the authors themselves. Articles broadly examining patient experiences of genetic testing were excluded.^14,15,16^

### Data Extraction

The final list of articles identified as eligible in the updated search was combined with the studies identified by Kohler et al^1^ for the purposes of data extraction and analysis. We extracted study data from all articles using Excel (Microsoft). One author (E.G.M.) first extracted verbatim text reporting key study and participant characteristics, including bibliographic information (eg, authors, date of publication, title), primary methodology (qualitative, quantitative, or other), number of participants by type (patient, family member, public, or other), and demographic characteristics, including race, ethnicity, gender (or sex), educational attainment, and income. Two independent reviewers (E.G.M., J.L.Y., A.R., and/or M.C.H.) coded the excerpted text using a prespecified coding scheme to create the final data set used for analysis. The complete codebook used for all variables is provided in eTable 3 in Supplement 1. The final complete data set used for analysis is provided in eTable 4 in Supplement 2.

The study team first reviewed the extracted text to develop a structured codebook designed to address variability in reporting across studies while maintaining current reporting standards (specifically, race and ethnicity^17^ and sex or gender^18^) whenever possible. However, given that current standards are not often reflected in previously published literature, a number of adaptations were required in order to report the data. For example, although current guidelines recommend specifying how gender was assessed (eg, self-report or observation), this information was not present in included studies.

As the terms *sex* and *gender* were used interchangeably in many of the articles in our review, we were unable to disaggregate these in our analysis and therefore use the terms *men* or *male* and *women* or *female* in an effort to be as specific and inclusive as possible. In addition, a number of studies reported only 1 category for sex or gender (eg, “75% women”). Because the original reporting assumed binary gender, we coded the remaining participants in these studies as men or women (ie, if reported as “75% women,” the remainder were coded as men). Nonbinary, transgender, or queer identities were categorized separately whenever reported in the original studies. We refer to this variable in our own reporting as “sex or gender” to clearly acknowledge this distinction.

Data on both race and ethnicity were recorded separately when provided. If only 1 category was reported (eg, “75% White”), data on race and ethnicity for the remaining participants were categorized as missing. If the original studies allowed participants to report multiple categories, all data were included in summary reporting (eg, individuals were coded as both White and Hispanic when reported in a single category) and accounted for in data analysis as described next. We refer to this variable in our own reporting as “race and ethnicity,” as we have listed both types of demographics together. eTable 2 in Supplement 1 provides a detailed list of the terms consolidated under each category used for sex or gender, race and ethnicity, and education.

Data on income were typically presented categorically, although the income ranges used varied widely across studies. Income data were therefore consolidated into binary categories of above or below the US median income of approximately $67 000. Participants in categories that crossed the median (eg, $40 000-$80 000) were distributed proportionally to the distribution for each income range in the US population.^19^

We had planned to include studies conducted in any location, but variation in terminology used for reporting demographics in different countries made synthesis impossible, and our final sample included only studies conducted with US participants. Because a goal of this review was to assess the generalizability of the available literature—itself an element of quality—and not the validity of the study results themselves, a broader quality analysis was not deemed relevant.

### Statistical Analysis

To summarize data across studies, we calculated the proportion of participants in each demographic category in each study. We then calculated the mean (SD) of these values across all studies, for each study method (qualitative, quantitative), and for each participant type (patient, family member, public). We also calculated these summary statistics for studies with the potential to skew the demographics, including those focused on conditions disproportionately affecting a particular sex or gender (eg, breast cancer) and those focused specifically on racial and ethnic diversity.

## Results

Our updated search identified 747 studies across the 4 databases used. After 316 duplicates were removed, 431 studies were screened for eligibility based on title and abstract content. This process identified 357 studies that were clearly ineligible. Of the remaining 74 studies, 28 were excluded because they lacked empirical data (15 studies); did not directly address utility (10 studies); included only participants other than patients, families, or the public (1 study); or were additional duplicates (2 studies). We then combined these 46 newly identified studies with the 31 studies included in the review by Kohler et al.^1^ Finally, due to the aforementioned challenges in data synthesis, we removed 25 studies conducted internationally, for a final sample of 52 studies (Figure).^4,5,6,7,8,20,21,22,23,24,25,26,27,28,29,30,31,32,33,34,35,36,37,38,39,40,41,42,43,44,45,46,47,48,49,50,51,52,53,54,55,56,57,58,59,60,61,62,63,64,65,66^

**Figure.**
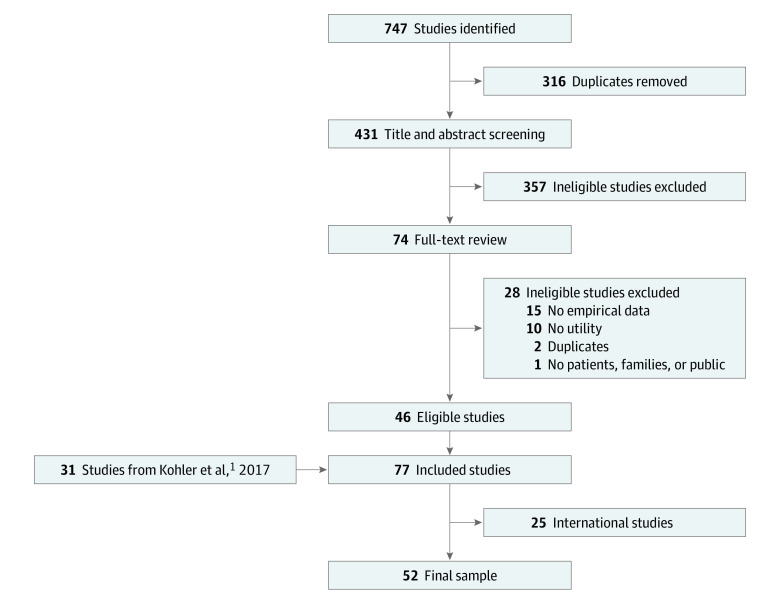
Study Flow Diagram

The 52 eligible studies included 13 251 eligible participants (Table 1). Of these studies, 31 (59.6%) used primarily qualitative methodology, although they accounted for only 1813 participants (13.7%) across all studies. Nearly half of the studies (24 [46.2%]) included only patients, 14 (26.9%) included only family members, 7 (13.5%) included only members of the public, and 7 (13.5%) included multiple participant types.

**Table 1.** Study Characteristics and Demographic Data Reporting^a^

Characteristic	Included studies^b^	Included participants
Primary methodology		
Qualitative	31 (59.6)	1813 (13.7)
Quantitative	19 (36.5)	11 392 (86.0)
Other	2 (3.8)	46 (0.3)
Participant type		
Patients	24 (46.2)	5894 (44.5)
Family members	14 (26.9)	1652 (12.5)
Public	7 (13.5)	5705 (43.1)
Multiple^c^	7 (13.5)	NA
Demographic reported		
Sex or gender		
Reported	48 (92.3)	12 660 (95.5)
Missing	4 (7.7)	591 (4.5)
Race and ethnicity^d^		
Reported	40 (76.9)	10 984 (82.9)
Missing	12 (23.1)	2652 (20.0)
Education		
Reported	38 (73.1)	10 974 (82.8)
Missing	14 (26.9)	2277 (17.2)
Income		
Reported	26 (50.0)	8836 (66.7)
Missing	26 (50.0)	4415 (33.3)
Total	52 (100)	13 251 (100)

Abbreviation: NA, not applicable.

^a^ Data are expressed as No. (%) of included studies or participants.

^b^ The median year of publication for included studies was 2017 (IQR, 2013-2019).

^c^ Multiple participant types were included.

^d^ Participants could select more than 1 category, so totals exceed 100%.

### Sex or Gender

Gender was the most frequently reported demographic variable, reported in 48 included studies (92.3%) for 12 660 participants (95.5%) across all studies (Table 1). Only 2 studies included participants who reported a nonbinary gender (n = 3). The mean (SD) proportion of women or female participants across all studies was 70.8% (20.5%). When studies with a focus on women or females were excluded, the mean (SD) proportion of women decreased only slightly in the remaining studies (65.3% [17.7%]). The mean (SD) proportion of women or female participants was somewhat higher in qualitative studies (78.4% [20.2%]) and in studies that included only family members (79.5% [14.3%]) (Table 2).

**Table 2.** Distribution of Demographic Characteristics Across Studies^a^

Characteristic	All, proportion of participants	Study method			Study participants			Excludes studies focused on	
		Qualitative	Quantitative	Patients		Family	Public	Women	Diversity
Gender or sex, No. of participants	12 660	1700	10 917	4025		1539	4493	575	71
Men or male	29.2 (20.5)	25.2 (20.2)	35.8 (18.9)	31.9 (23.8)		20.5 (14.3)	44.6 (14.7)	34.7 (17.7)	29.6 (20.8)
All other categories	0 (0.1)	0	0 (0.1)	0		0	0.1 (0.2)	0	0
Women or female	70.8 (20.5)	78.4 (20.2)	64.2 (18.9)	68.1 (23.8)		79.5 (14.3)	14.3 (14.8)	65.3 (17.7)	70.4 (20.8)
Race and ethnicity, No. of participants^b^	10 984	1500	9444	3660		1518	3041	565	71
American Indian or Indigenous	0.1 (0.7)	0	1.4 (1.1)	0 (0.2)		0 (0.2)	0	0.1 (0.7)	0.1 (0.7)
Asian or Pacific Islander	4.3 (5.2)	4.5 (5.9)	4.2 (3.9)	5.8 (6.1)		3.1 (4.3)	3.6 (2.9)	4.5 (5.2)	4.2 (5.1)
Black	7.5 (17.1)	8.6 (8.5)	2.9 (2.8)	6.4 (11.1)		9.7 (26.3)	4.8 (6.4)	8.4 (18.2)	5.2 (8.3)
Hispanic or Latinx	7.6 (12.1)	8.5 (12.9)	6.3 (11.0)	5.6 (9.0)		11.9 (17.2)	4.1 (3.6)	8.5 (12.7)	6.9 (10.8)
White	76.1 (22.0)	73.9 (24.8)	81.9 (14.8)	78.5 (16.3)		68.4 (29.1)	85.3 (10.8)	74.2 (22.5)	79.2 (17.3)
Multiple races	0.6 (1.6)	0.8 (1.9)	0.2 (0.7)	0		0.9 (2.2)	1.1 (1.5)	0.6 (1.7)	0.4 (1.3)
Other	3.8 (6.9)	3.8 (8.0)	4.1 (4.6)	3.7 (6.8)		6.0 (8.7)	1.1 (2.1)	3.6 (6.5)	4.0 (7.0)
Education, No. of participants	10 974	1156	9778	3172		1092	4457	27	71
Less than a college degree	35.5 (19.9)	37.8 (17.1)	32.4 (24.2)	28.5 (21.9)		43.1 (17.0)	35.7 (27.0)	36.0 (19.9)	33.9 (19.0)
College degree or higher	64.5 (19.9)	62.2 (17.1)	67.6 (24.2)	71.5 (21.9)		56.9 (17.0)	64.3 (27.0)	64.0 (19.9)	66.1 (19.0)
Income, No. of participants	8836	888	7908	2729		738	3601	25	71
Below median	32.6 (19.2)	35.6 (21.3)	30.8 (17.0)	22.9 (16.0)		45.4 (20.8)	40.5 (21.7)	33.7 (18.8)	30.7 (17.5)
Above median	67.4 (19.2)	64.4 (21.3)	69.2 (17.0)	77.1 (16.0)		54.6 (20.8)	59.5 (21.7)	66.2 (18.3)	69.3 (17.5)

^a^ Unless indicated otherwise, data are presented as the mean (SD) proportion.

^b^ Because participants could select more than 1 category, totals may exceed 100%.

### Race and Ethnicity

Race and ethnicity data were provided in 40 studies (76.9%) for 10 984 participants (82.9%) across studies (Table 1). Six studies allowed participants to choose more than 1 category of race or ethnicity. The mean (SD) proportions of participants by race and ethnicity were as follows: 0.1% (0.7%) were American Indian or Indigenous, 4.3% (5.2%) were Asian or Pacific Islander, 7.5% (17.1%) were Black, 7.6% (12.1%) were Hispanic or Latinx, 76.1% (22.0%) were White, 0.6% (1.6%) were of multiple races, and 3.8% (6.9%) were of other race or ethnicity. When the 2 studies focused on diversity were removed, the mean (SD) proportion of White participants in the remaining studies increased further (79.2% [17.3%]). On average, studies with qualitative methodologies and family member participants were somewhat more diverse, with proportions of 73.9% (24.8%) and 68.4% (29.1%) White, respectively (Table 2). In reviewing the data, we noted that there were only 274 Black participants in any eligible quantitative study of utility compared with 7950 White participants. Further, 41 of the 98 Black participants (41.8%) in all qualitative studies came from a single study specifically focused on the perspectives of Black patients.^38^

### Education

Education level was reported in 38 studies (73.1%) for 10 974 participants (82.8%) (Table 1). The mean (SD) proportion of participants with a college degree or higher was 64.5% (19.9%) across all studies (Table 2). This mean (SD) proportion was even larger in studies with only patients (71.5% [21.9%]) but was lower in studies with only family members (56.9% [17.0%]) (Table 2).

### Income

Income was reported in only 26 studies (50.0%) for 8836 participants (66.7%). The mean (SD) proportion of participants reporting income above the US median income was 67.4% (19.2%). This mean (SD) proportion was higher in quantitative studies (69.2% [17.0%]) and in studies with only patients (77.1% [16.0%]), while studies involving only family members reported just over half (54.6% [20.8%]) above the median income.

## Discussion

The results of this systematic review suggest that the literature on the personal utility of genetic and genomic testing in the US has disproportionately captured the perspectives of a relatively narrow subset of the population—specifically White, college-educated women with above-average household income. These findings are particularly concerning given recent research suggesting important differences in the perceived utility of genetic and genomic tests across participants with diverse backgrounds.^8^ These data also are not representative of the US population. For example, only 37.9% of adults in the US have a bachelor degree, only 61% identify as White, approximately half identify as women, and 50% have income status below the median.^67^

The presentation of the findings from the included studies also is notable. As described earlier, nearly half of all Black participants in qualitative studies came from a single study specifically focused on the perspectives of Black patients.^38^ This study explicated its focus on Black perspectives in its title. However, many of the other studies we reviewed, which included all or nearly all White participants, did not state an explicit focus on understanding the perspectives of White patients. Such patterns only serve to normalize and reinforce narratives prioritizing White perspectives.

Further, information on race and ethnicity, education, and income was either not reported or missing for a large proportion of study participants. While the increasing use of standards for reporting these data will hopefully rectify this issue in the future,^17,68^ achieving systematic reporting across all journals will take time. Given the potential for such demographic characteristics to influence participant perspectives on the personal utility of genetics and genomics, future studies should ensure robust and systematic collection of participants’ demographic characteristics.

The need to diversify genomic data sets has been widely recognized in recent years,^9^ but diverse participants continue to be underrepresented in genetic and genomic testing in both clinical and research contexts. The existing literature on barriers has focused on characteristics of the participants themselves, such as cultural beliefs, lack of trust in research due to past transgressions, privacy concerns, low health literacy, and financial and insurance barriers.^69,70,71,72,73^ An additional barrier may include a relative lack of perceived utility of these tests, as suggested in a recent study.^8^ These preliminary findings require further examination to confirm and, if indicated, to identify the underlying drivers of such differences. These could include differences among diverse patients in terms of their perspectives on the potential value of genetics and genomics, their attitudes and beliefs regarding these tests, and/or their ability to actualize the potential benefits of this testing due to language, financial, or other barriers. Inclusion of diverse participants in studies such as these is an essential first step to answering these questions.

### Limitations

Our systematic review has a number of limitations. As noted in the eligibility criteria, the term *utility* takes on many different meanings depending on one’s disciplinary orientation. In these varied contexts, other terms (eg, *preferences*, in the context of health economics) may be used interchangeably with the concept of utility. It is possible that we did not include the full range of studies in all disciplines that incorporate the concept of utility. In addition, as the boundaries of utility itself are vague, our requirement that the eligible studies include the term *utility* may have excluded studies that are otherwise conceptually relevant. In addition, our review was limited to studies including US participants and therefore cannot provide insights for international contexts. Finally, due to the narrow scope of this study, we did not conduct a comprehensive risk-of-bias assessment due to missing results or a formal assessment of certainty.

## Conclusion

The results of this systematic review suggest that our understanding of the personal utility of genetics and genomics is disproportionately based on the perspectives of White women with a college education and above-average income. To achieve the National Human Genome Research Institute goal of “equitable use of genomics in healthcare that avoids exacerbating and strives towards reducing health disparities,”^74^ we need to both collect diverse genomic data and better understand diverse perspectives on the value of these data.

## Notes

Supplement 1. **eTable 1.** Search Strings **eTable 2.** Synthesis of Demographic Categories **eTable 3.** Complete Codebook

Supplement 2. **eTable 4.** Complete Dataset

Supplement 3.
Data Sharing Statement
