# Supplementary material for: Demographic Characteristics Associated With Perceptions of Personal Utility in Genetic and Genomic Testing: A Systematic Review
Source: JAMA Netw Open. 2023 May 5;6(5):e2310367. doi: 10.1001/jamanetworkopen.2023.10367 (PMC10163389; doi:10.1001/jamanetworkopen.2023.10367)
Supplement: Supplement 1. — eTable 1. Search Strings eTable 2. Synthesis of Demographic Categories eTable 3. Complete Codebook [file jamanetwopen-e2310367-s001.pdf]

Supplementary Online Content

Miller EG, Young JL, Rao A, Ward-Lev E, Halley MC. Demographic characteristics associated with perceptions of personal utility in genetic and genomic testing: a systematic review. *JAMA Netw Open*. 2023;6(5):e2310367. doi:10.1001/jamanetworkopen.2023.10367

eTable 1. Search Strings
eTable 2. Synthesis of Demographic Categories
eTable 3. Complete Codebook

This supplementary material has been provided by the authors to give readers additional information about their work.

© 2023 Miller EG et al. *JAMA Network Open*.

eTable 1. Search Strings

PubMed
**Search String 1:** ("genetic testing"[majr] OR "genetic testing"[tiab] OR "genetics testing"[tiab] OR "genome testing"[tiab] OR "genomic testing"[tiab] OR "genomics testing"[tiab] OR "genetic sequencing"[tiab] OR "genetics sequencing"[tiab] OR "genome sequencing"[tiab] OR "genomic sequencing"[tiab] OR "genomics sequencing"[tiab] OR "exome sequencing"[tiab] OR "exomic sequencing"[tiab] OR "sequence analysis, dna"[majr] OR "genetic profiling"[tiab] OR "genome profiling"[tiab] OR "genomic profiling"[tiab] OR "genomics profiling"[tiab] OR "direct to consumer testing"[tiab] OR "direct to consumer genetic"[tiab] OR "direct to consumer genetics"[tiab] OR "direct to consumer genome"[tiab] OR "direct to consumer genomic"[tiab] OR "direct to consumer genomics"[tiab] OR "personal genetic"[tiab] OR "personal genetics"[tiab] OR "personal genome"[tiab] OR "personal genomic"[tiab] OR "personal genomics"[tiab] OR "individual genetic"[tiab] OR "individual genetics"[tiab] OR "individual genome"[tiab] OR "individual genomic"[tiab] OR "individual genomics"[tiab] OR "genetic susceptibility testing"[tiab] OR "genomic risk profiling"[tiab] OR "genome based testing"[tiab]) AND ("personal utility"[tiab] OR "patient utility"[tiab] OR "individual utility"[tiab] OR "participant utility"[tiab] OR "consumer utility"[tiab] OR "public utility"[tiab] OR "health utility"[tiab] OR "health-related utility"[tiab] OR “perceived utility”[tiab] OR “patient-oriented utility”[tiab] OR "personal meaning"[tiab] OR "patient meaning"[tiab] OR "individual meaning"[tiab] OR "participant meaning"[tiab] OR "consumer meaning"[tiab] OR "public meaning"[tiab] OR "personal value"[tiab] OR "patient value"[tiab] OR "individual value"[tiab] OR "participant value"[tiab] OR "consumer value"[tiab] OR "public value"[tiab] OR "personal benefit"[tiab] OR "personal benefits"[tiab] OR "personal harm"[tiab] OR "personal harms"[tiab] OR "personal outcome"[tiab] OR "personal outcomes"[tiab] OR "non-clinical benefit"[tiab] OR "non-clinical benefits"[tiab] OR "nonclinical benefit"[tiab] OR "nonclinical benefits"[tiab] OR "non-medical benefit"[tiab] OR "non-medical benefits"[tiab] OR "nonmedical benefit"[tiab] OR "nonmedical benefits"[tiab] OR “psychological outcome”[tiab] OR “psychological impact”[tiab] OR “psychological outcomes”[tiab] OR “psychological impacts”[tiab])
**Search String 2:** ("genetic testing"[majr] OR "genetic testing"[tiab] OR "genetics testing"[tiab] OR "genome testing"[tiab] OR "genomic testing"[tiab] OR "genomics testing"[tiab] OR "genetic sequencing"[tiab] OR "genetics sequencing"[tiab] OR "genome sequencing"[tiab] OR "genomic sequencing"[tiab] OR "genomics sequencing"[tiab] OR "exome sequencing"[tiab] OR "exomic sequencing"[tiab] OR "sequence analysis, dna"[majr] OR "genetic profiling"[tiab] OR "genome profiling"[tiab] OR "genomic profiling"[tiab] OR "genomics profiling"[tiab] OR "direct to consumer testing"[tiab] OR "direct to consumer genetic"[tiab] OR "direct to consumer genetics"[tiab] OR "direct to consumer genome"[tiab] OR "direct to consumer genomic"[tiab] OR "direct to consumer genomics"[tiab] OR "personal genetic"[tiab] OR "personal genetics"[tiab] OR "personal genome"[tiab] OR "personal genomic"[tiab] OR "personal genomics"[tiab] OR "individual genetic"[tiab] OR "individual genetics"[tiab] OR "individual genome"[tiab] OR "individual genomic"[tiab] OR "individual genomics"[tiab] OR "genetic susceptibility testing"[tiab] OR "genomic risk profiling"[tiab] OR "genome based testing"[tiab]) AND ((personal[ti] OR patient[ti] OR individual[ti] OR participant[ti] OR consumer[ti]) AND (utility[ti] OR meaning[ti] OR value[ti] OR benefit[ti])))
Scopus
**Search String 1:** TITLE ("genetic testing" OR "genetics testing" OR "genome testing" OR "genomic testing" OR "genomics testing" OR "genetic sequencing" OR "genetics sequencing" OR "genome sequencing" OR "genomic sequencing" OR "genomics sequencing" OR "exome sequencing" OR "exomic sequencing" OR "sequence analysis" OR "genetic profiling" OR "genome profiling" OR "genomic profiling" OR "genomics profiling" OR "direct to consumer testing" OR "direct to consumer genetic" OR "direct to consumer genetics" OR "direct to consumer genome" OR "direct to consumer genomic" OR "direct to consumer genomics" OR "personal genetic" OR "personal genetics" OR "personal genome" OR "personal genomic" OR "personal genomics" OR "individual genetic" OR "individual genetics" OR "individual genome" OR "individual genomic" OR "individual genomics" OR "genetic susceptibility testing" OR "genomic risk profiling" OR "genome based testing") AND TITLE-ABS-KEY ("personal utility" OR "patient utility" OR "individual utility" OR "participant utility" OR "consumer utility" OR "public utility" OR "health utility" OR "health-related utility" OR “perceived utility” OR “patient-oriented utility” OR "personal meaning" OR "patient meaning" OR "individual meaning" OR "participant meaning" OR "consumer meaning" OR "public meaning"

© 2023 Miller EG et al. *JAMA Network Open*.

OR "clinical meaning" OR "medical meaning" OR "personal value" OR "patient value" OR "individual value" OR "participant value" OR "consumer value" OR "public value" OR “psychological outcome” OR “psychological impact” OR “psychological outcomes” OR “psychological impacts” OR "personal benefit" OR "personal benefits" OR "personal harm" OR "personal harms" OR "personal outcome" OR "personal outcomes" OR "non-clinical benefit" OR "non-clinical benefits" OR "nonclinical benefit" OR "nonclinical benefits" OR "non-medical benefit" OR "non-medical benefits" OR "nonmedical benefit" OR "nonmedical benefits") AND (LIMIT-TO (LANGUAGE , "English"))
**Search String 2:** (TITLE ("genetic testing" OR "genetics testing" OR "genome testing" OR "genomic testing" OR "genomics testing" OR "genetic sequencing" OR "genetics sequencing" OR "genome sequencing" OR "genomic sequencing" OR "genomics sequencing" OR "exome sequencing" OR "exomic sequencing" OR "sequence analysis" OR "genetic profiling" OR "genome profiling" OR "genomic profiling" OR "genomics profiling" OR "direct to consumer testing" OR "direct to consumer genetic" OR "direct to consumer genetics" OR "direct to consumer genome" OR "direct to consumer genomic" OR "direct to consumer genomics" OR "personal genetic" OR "personal genetics" OR "personal genome" OR "personal genomic" OR "personal genomics" OR "individual genetic" OR "individual genetics" OR "individual genome" OR "individual genomic" OR "individual genomics" OR "genetic susceptibility testing" OR "genomic risk profiling" OR "genome based testing")) AND (TITLE (personal OR patient OR individual OR participant OR consumer) AND TITLE (utility OR meaning OR value OR benefit))
Web of Science
**Search String 1:** TITLE: ("genetic testing" OR "genetics testing" OR "genome testing" OR "genomic testing" OR "genomics testing" OR "genetic sequencing" OR "genetics sequencing" OR "genome sequencing" OR "genomic sequencing" OR "genomics sequencing" OR "exome sequencing" OR "exomic sequencing" OR "sequence analysis" OR "genetic profiling" OR "genome profiling" OR "genomic profiling" OR "genomics profiling" OR "direct to consumer testing" OR "direct to consumer genetic" OR "direct to consumer genetics" OR "direct to consumer genome" OR "direct to consumer genomic" OR "direct to consumer genomics" OR "personal genetic" OR "personal genetics" OR "personal genome" OR "personal genomic" OR "personal genomics" OR "individual genetic" OR "individual genetics" OR "individual genome" OR "individual genomic" OR "individual genomics" OR "genetic susceptibility testing" OR "genomic risk profiling" OR "genome based testing") *AND* TOPIC: ("personal utility" OR "patient utility" OR "individual utility" OR "participant utility" OR "consumer utility" OR "public utility" OR "health utility" OR "health-related utility" OR “perceived utility” OR “patient-oriented utility” OR "personal meaning" OR "patient meaning" OR "individual meaning" OR "participant meaning" OR "consumer meaning" OR "public meaning" OR “psychological outcome” OR “psychological impact” OR “psychological outcomes” OR “psychological impacts” OR "personal value" OR "patient value" OR "individual value" OR "participant value" OR "consumer value" OR "public value" OR "clinical value" OR "medical value" OR "personal benefit" OR "personal benefits" OR "personal harm" OR "personal harms" OR "personal outcome" OR "personal outcomes" OR "non-clinical benefit" OR "non-clinical benefits" OR "nonclinical benefit" OR "nonclinical benefits" OR "non-medical benefit" OR "non-medical benefits" OR "nonmedical benefit" OR "nonmedical benefits")<br>Refined by:LANGUAGES: (ENGLISH)
**Search String 2:** TITLE: ("genetic testing" OR "genetics testing" OR "genome testing" OR "genomic testing" OR "genomics testing" OR "genetic sequencing" OR "genetics sequencing" OR "genome sequencing" OR "genomic sequencing" OR "genomics sequencing" OR "exome sequencing" OR "exomic sequencing" OR "sequence analysis" OR "genetic profiling" OR "genome profiling" OR "genomic profiling" OR "genomics profiling" OR "direct to consumer testing" OR "direct to consumer genetic" OR "direct to consumer genetics" OR "direct to consumer genome" OR "direct to consumer genomic" OR "direct to consumer genomics" OR "personal genetic" OR "personal genetics" OR "personal genome" OR "personal genomic" OR "personal genomics" OR "individual genetic" OR "individual genetics" OR "individual genome" OR "individual genomic" OR "individual genomics" OR "genetic susceptibility testing" OR "genomic risk profiling" OR "genome based testing") *AND* TITLE: (((personal OR patient OR individual OR participant OR consumer) AND (utility OR meaning OR value OR benefit))) Refined by:LANGUAGES: (ENGLISH)
Embase

© 2023 Miller EG et al. *JAMA Network Open*.

**Search String 1:** ('genetic screening'/exp/mj OR 'genetic testing':ti OR 'genetics testing':ti OR 'genome testing':ti OR 'genomic testing':ti OR 'genomics testing':ti OR 'genetic sequencing':ti OR 'genetics sequencing':ti OR 'genome sequencing':ti OR 'genomic sequencing':ti OR 'genomics sequencing':ti OR 'exome sequencing':ti OR 'exomic sequencing':ti OR 'sequence analysis'/exp/mj OR 'sequence analysis':ti OR 'genetic profiling':ti OR 'genome profiling':ti OR 'genomic profiling':ti OR 'genomics profiling':ti OR 'direct to consumer testing':ti OR 'direct to consumer genetic':ti OR 'direct to consumer genetics':ti OR 'direct to consumer genome':ti OR 'direct to consumer genomic':ti OR 'direct to consumer genomics':ti OR 'personal genetic':ti OR 'personal genetics':ti OR 'personal genome':ti OR 'personal genomic':ti OR 'personal genomics':ti OR 'individual genetic':ti OR 'individual genetics':ti OR 'individual genome':ti OR 'individual genomic':ti OR 'individual genomics':ti OR 'genetic susceptibility testing':ti OR 'genomic risk profiling':ti OR 'genome based testing':ti) AND ('personal utility':ab,ti OR 'patient utility':ab,ti OR 'individual utility':ab,ti OR 'participant utility':ab,ti OR 'consumer utility':ab,ti OR 'public utility':ab,ti OR 'health utility':ab,ti OR 'health-related utility':ab,ti OR 'perceived utility':t,ab OR 'patient-oriented utility':ti,ab OR 'personal meaning':ab,ti OR 'patient meaning':ab,ti OR 'individual meaning':ab,ti OR 'participant meaning':ab,ti OR 'consumer meaning':ab,ti OR 'public meaning':ab,ti OR 'clinical meaning':ab,ti OR 'medical meaning':ab,ti OR 'personal value':ab,ti OR 'patient value':ab,ti OR 'individual value':ab,ti OR 'participant value':ab,ti OR 'consumer value':ab,ti OR 'public value':ab,ti OR 'psychological outcome':ab,ti OR 'psychological impact':ab,ti OR 'psychological outcomes':ab,ti OR 'psychological impacts':ab,ti OR 'personal benefit':ab,ti OR 'personal benefits':ab,ti OR 'personal harm':ab,ti OR 'personal harms':ab,ti OR 'personal outcome':ab,ti OR 'personal outcomes':ab,ti OR 'non-clinical benefit':ab,ti OR 'non-clinical benefits':ab,ti OR 'nonclinical benefit':ab,ti OR 'nonclinical benefits':ab,ti OR 'non-medical benefit':ab,ti OR 'non-medical benefits':ab,ti OR 'nonmedical benefit':ab,ti OR 'nonmedical benefits':ab,ti) AND ([english]/lim AND ([embase]/lim OR [embase classic]/lim)))
**Search String 2:** (('genetic screening'/exp/mj OR 'genetic testing':ti OR 'genetics testing':ti OR 'genome testing':ti OR 'genomic testing':ti OR 'genomics testing':ti OR 'genetic sequencing':ti OR 'genetics sequencing':ti OR 'genome sequencing':ti OR 'genomic sequencing':ti OR 'genomics sequencing':ti OR 'exome sequencing':ti OR 'exomic sequencing':ti OR 'sequence analysis'/exp/mj OR 'sequence analysis':ti OR 'genetic profiling':ti OR 'genome profiling':ti OR 'genomic profiling':ti OR 'genomics profiling':ti OR 'direct to consumer testing':ti OR 'direct to consumer genetic':ti OR 'direct to consumer genetics':ti OR 'direct to consumer genome':ti OR 'direct to consumer genomic':ti OR 'direct to consumer genomics':ti OR 'personal genetic':ti OR 'personal genetics':ti OR 'personal genome':ti OR 'personal genomic':ti OR 'personal genomics':ti OR 'individual genetic':ti OR 'individual genetics':ti OR 'individual genome':ti OR 'individual genomic':ti OR 'individual genomics':ti OR 'genetic susceptibility testing':ti OR 'genomic risk profiling':ti OR 'genome based testing':ti) AND (personal:ti OR patient:ti OR individual:ti OR participant:ti OR consumer:ti AND (utility:ti OR meaning:ti OR value:ti OR benefit:ti) AND ([english]/lim AND ([embase]/lim OR [embase classic]/lim)))

© 2023 Miller EG et al. *JAMA Network Open*.

eTable 2. Synthesis of Demographic Categories

A. Sex or Gender	
**Coded Categories**	**Original Terms in Manuscripts***
Men or Male	• Male<br>• Man<br>• Boy
Other Categories	• Nonbinary<br>• Transgender Male<br>• Transgender Female<br>• Other sex or gender
Women or Female	• Female<br>• Woman<br>• Girl

*These terms are quoted directly from the original manuscripts and therefore do not necessarily comply with current reporting standards.

B. Race and Ethnicity	
**Coded Categories**	**Original Terms in Manuscripts***
Asian American or Pacific Islander (AAPI)	• Asian<br>• Asian and not Hispanic or Latino<br>• South Asian<br>• East Asian<br>• Asian American<br>• Native Hawaiian<br>• Pacific Islander<br>• Asian/Pacific Islander
Black	• Black<br>• African American<br>• African American and not Hispanic or Latino<br>• Non-Hispanic Black<br>• Sub-Saharan African
Hispanic/Latino	• Hispanic<br>• Latinx<br>• White/Hispanic

© 2023 Miller EG et al. *JAMA Network Open*.

B. Race and Ethnicity	
	• Latino<br>• Hispanic White<br>• Hispanic or Latino
Multiracial	• Mixed ethnicity<br>• Mixed<br>• Multiethnic/multiracial<br>• Hispanic Black<br>• Hispanic Asian
Native American or Indigenous	• American Indian<br>• Native American<br>• Alaskan Native
Other	• Other race or ethnicity<br>• Non-Hispanic or Latino, Other Race<br>• West Indian<br>• East Indian<br>• Caribbean/South American<br>• Ashkenazi Jewish
White	• White<br>• Caucasian<br>• White/Non-Hispanic<br>• Non-Latino White<br>• White and not Hispanic or Latino<br>• Western European<br>• Other Mixed Caucasian<br>• Southern European<br>• Northern European

*These terms are quoted directly from the original manuscripts and therefore do not necessarily comply with current reporting standards.

C. Education	
**Coded Categories**	**Original Terms in Manuscripts***
Less than College Degree: High School or Less	• Some primary or elementary school, or completion<br>• Some high school or secondary school, or high/secondary school degree, or GED
Less than College Degree: Some College	• Some community college or community college degree<br>• Vocational training

© 2023 Miller EG et al. *JAMA Network Open*.

C. **Education**	
	• Two-year college or associate's degree<br>• Some technical school or technical school degree<br>• Some college, some university
Less than College Degree: Unspecified	• Less than a college degree<br>• Greater than high school degree
College Degree or Higher: Unspecified	• Bachelor's degree or higher<br>• College, advanced or professional degree
College Degree or Higher: Bachelor's Degree	• College or university degree<br>• Bachelor's degree
College Degree or Higher: Graduate Degree	• Master's degree<br>• PhD, MD, other advance coursework or postgraduate degree

*These terms are quoted directly from the original manuscripts and therefore do not necessarily comply with current reporting standards.

© 2023 Miller EG et al. *JAMA Network Open*.

eTable 3. Complete Codebook

Variable Name	Variable Type	Definition	Codes *If applicable*
UPI	Text	Assigned unique identifier	
DOI	Text	Digital Object Identifier	
Title	Text	Study Title	
Journal	Text	Journal Name or Abbreviation	
Year	Text	Year of Publication	
N_Study	Numeric	Total number of all types of participants in the study.	
N_Patient	Numeric	Total number of individuals identified as patients in the study.	
N_Family	Numeric	Total number of individuals identified as family members of patients or caregivers in the study. Include here "advocates" or other individuals whose opinions are collected as a proxy for patients. Do not include individuals recruited as members of the general public who happen to be parents.	
N_Public	Numeric	Total number of individuals identified as members of the general public.	
N_Ineligible	Numeric	Total number of individuals in the study who are not eligible (e.g., physicians, policymakers)	
N_Eligible	Numeric	Total number of patients, family members, or public included in the study.	
Method_Type	Categorical	Indicates whether the paper primarily reporting on a quantitative or a qualitative method. Qualitative papers tended to be exploratory, small samples and involve interviews, focus groups, or case studies. Quantitative papers tended to use surveys and have larger sample sizes. If both methods were used, primary method determined by consensus review by the study team. For example, a study that included a sample in which all participants were interviewed and also complete a survey was characterized as qualitative. If all participants complete a survey and only a small subset are interviewed, then it is characterized as a quantitative. Other was used to categorize infrequently used methodologies (e.g., Delphi methods).	1 = Quantitative<br>2 = Qualitative<br>3 = Other
Edu_Rep	Binary	Indicates whether the paper presents data on educational attainment for eligible participants.	1=Yes<br>0=No
N_HS_OrLess	Numeric	Number of participants categorized as having completed a high school degree or less.	
N_Some_College	Numeric	Number of participants categorized as having completing education beyond high school but not completed a four-year college degree.	

© 2023 Miller EG et al. *JAMA Network Open*.

Variable Name	Variable Type	Definition	Codes *If applicable*
N_LessThan_CollegeDeg_Unspec	Numeric	Number of participants categorized as having not completed a four-year college degree. See Supplementary Appendix 2 for details. Only use if data not disaggregated by N_HS_OrLess and N_Some_College.	
N_CollegeDeg_OrMore_Unspec	Numeric	Number of participants categorized as having completed a four-year college degree or more education. See Supplementary Appendix 2 for details. Only use if data not disaggregated by level of degree	
N_Bachelor_Deg	Numeric	Number of participants categorized as having completed a four-year college degree.	
N_Graduate_Deg	Numeric	Number of participants categorized as having completed a graduate degree.	
N_Edu_Miss	Numeric	Number of participants for whom education data is missing, even though study does report education in general. Includes missing, not reported, or declined. Also includes undefined "other" category if used.	
N_Edu_Rep_Total	Numeric	Total number of individuals with reported education data (does not include those with missing data).	
N_ Inc_Rep	Binary	Indicates whether the paper reports data on income for eligible participants.	1=Yes<br>0=No
N_Inc_Miss	Numeric	Number of participants for whom income data is missing, even though study does report income in general. Includes missing, not reported, or declined.	
N_Inc_BelowMedian	Numeric	Number of participants with income below the U.S. median. Calculated using US population income distribution.	
N_Inc_AboveMedian	Numeric	Number of participants with income below the U.S. median. Calculated using US population income distribution.	
N_Inc_Rep	Numeric	Total number of participants for with income data reported (does not include those with missing data).	
Gen_Rep	Binary	Indicates whether the paper reports data on sex or gender for eligible participants.	1=Yes<br>0=No
Gender_Focus	Binary	Indicates whether the paper focuses on (and only recruit) females or women. Includes studies on breast cancer and prenatal testing.	1=Yes<br>0=No
N_Women	Numeric	Number of participants categorized as women or female.	
N_Men	Numeric	Number of participants categorized as men or male.	
N_Gen_Other	Numeric	Number of participants self-reporting a gender other than man or woman.	
N_Gen_Miss	Numeric	Number of participants for whom data on sex and gender is missing. Includes missing, not reported, or declined.	

© 2023 Miller EG et al. *JAMA Network Open*.

Variable Name	Variable Type	Definition	**Codes**<br>*If applicable*
N_Gen_Rep_Total	Numeric	Total number of individuals with reported data on sex or gender (does not include those with missing data).	
Race_Rep	Binary	Indicates whether the paper reports race and/or ethnicity data. Do not treat country of origin as race or ethnicity if this is the only information provided.	1=Yes<br>0=No
Diversity_Focus	Binary	Indicates whether the study explicitly states a focus (and only recruit) individuals from diverse racial or eth.	1=Yes<br>0=No
N_NHWhite	Numeric	Number of participants categorized as non-Hispanic White.	
N_Black	Numeric	Number of participants categorized as Black.	
N_HispLatx	Numeric	Number of participants categorized as Hispanic or Latinx.	
N_AAPI	Numeric	Number of participants categorized as Asian-American or Pacific Islander.	
N_Native_Indig	Numeric	Number of participants categorized as Native American or another indigenous group.	
N_Multiracial	Numeric	Number of participants categorized as multiracial.	
N_Race_Other	Numeric	Number of participants categorized as another race or ethnicity.	
N_Race_Miss	Numeric	Number of participants for whom race and ethnicity data is reported as missing, even though study does report this information in general. Includes missing, not reported, or declined.	
N_Race_Rep_Total	Numeric	Total number of individuals with reported race and/or ethnicity data (does not include those with missing data).	
Race_Discrete	Binary	Indicates a study in which individuals were allowed to select multiple options for race and/or ethnicity.	1=Yes<br>0=No

© 2023 Miller EG et al. *JAMA Network Open*.
