# Supplementary material for: Demographic Characteristics Associated With Perceptions of Personal Utility in Genetic and Genomic Testing: A Systematic Review
Source: JAMA Netw Open. 2023 May 5;6(5):e2310367. doi: 10.1001/jamanetworkopen.2023.10367 (PMC10163389; doi:10.1001/jamanetworkopen.2023.10367)
Supplement: Supplement 3. — Data Sharing Statement [file jamanetwopen-e2310367-s003.pdf]

Data Sharing Statement

Miller. Demographic Characteristics Associated With Perceptions of Personal Utility in Genetic and Genomic Testing. *JAMA Netw Open*. Published May 05, 2023.
doi:10.1001/jamanetworkopen.2023.10367

Data
Data available: Yes
Data types: Data (not involving human participants)
How to access data: The final dataset and data dictionary can be accessed here: https://datadryad.org/stash/share/QfzdaoihOuHMabnOQOMywoY0nozZ1dnA6w5QH0ARpKA
When available: With publication

Supporting Documents
Document types: None

Additional Information
Who can access the data: Publicly available.
Types of analyses: Any purpose.
Mechanisms of data availability: Publicly available.
Any additional restrictions: None.
